# Immune Dysfunction as Measured by the Systemic Immune-Inflammation Index is Associated with the Sub-Type of Minimal Residual Disease and Outcome in Stage II Colon Cancer Treated with Surgery alone

**DOI:** 10.31557/APJCP.2021.22.8.2391

**Published:** 2021-08

**Authors:** Nigel P Murray, Ricardo Villalon, Shenda Orrego, Eghon Guzman

**Affiliations:** 1 *Faculty of Medicine, Finis Terrae University, Av. Pedro de Valdivia 1509, Providencia, Santiago, 7501015, Chile. *; 2 *Coloproctology Service, Hospital de Carabineros de Chile, Simón Bolívar 2200, Ñuñoa, Santiago, 7770199, Chile. *; 3 *Faculty of Medicine, University Mayor, San Pio X 2422, Providencia, Santiago, Chile. *

**Keywords:** Colon cancer, minimal residual disease, immune dysfunction, systemic inflammatory index, prognosis

## Abstract

**Objective:**

Within 5 years after curative surgery for stage II colon cancer 25% of patients will relapse due to minimal residual disease (MRD). MRD is the net result of the biological properties of subpopulations of primary tumour cells which enable them to disseminate, implant in distant tissues and survive and the immune system’s ability to eliminate them. We hypothesize that markers of immune dysfunction such as the systemic inflammation index (SII) are associated with the sub-type of MRD defined by bone marrow micro-metastasis (mM) and circulating tumour cells (CTCs). A higher immune dysfunction being associated with a more aggressive MRD and worse prognosis.

**Methods and Patients:**

Blood and bone marrow samples were taken to detect CTCs and mM using immunocytochemistry with anti-CEA one month after surgery. The SII, absolute neutrophil, platelet and lymphocyte counts (ANC, APC, ALC) were determined immediately pre-surgery and one month post-surgery. These were compared with the sub-types of MRD; Group I MRD (-); Group II mM positive and Group III CTC positive; cut-off values of SII of >700 and >900 were used. Follow-up was for up to 5 years or relapse and survival curves using Kaplan-Meier (KM) were calculated.

**Results:**

One hundred and eighty one patients (99 women) participated, mean age 68 years, median follow up 4.04 years; I: = 105 patients, II: N= 36 patients, III: N=40 patients. The SII significantly decreased post-surgery only in Group I patients. The frequency of SII >700 and >900 was significantly higher in Group III, between Groups I and II there was no significant difference. The SII was significantly associated with the number of CTCs detected. The 5-year KM was 98% Group I, 68% Group II and 7% Group III.

**Conclusions:**

The results of the study suggest that the severity of immune dysfunction as determined by the SII is associated with differing sub-types of MRD and a worse prognosis; increasing immune dysfunction is associated with a more aggressive CTC positive MRD sub-type; a more severe immune dysfunction is associated with a higher number of CTCs detected.

## Introduction

Colon cancer is the fourth most common cancer worldwide, representing 6.1% of all cancers and is the third commonest cause of cancer mortality in males and females (Bray et al., 2018). Stage II colon cancer as defined by the American Joint Committee on Cancer (8^th^ Edition) (Amin et al., 2017), is a heterogeneous disease and up to 25% of patients treated by surgery alone will develop metastasis (Engstrom et al., 2009). Stage II patients have been classified as low or high risk based on the pathological findings in the surgical specimen; high risk tumours have been classified as having one or more of the following features; stage pT4, poorly differentiated tumour; intestinal perforation; lympho-vascular / peri-neural invasion; a high number of lymph nodes examined and positive surgical margins (Provenzale et al., 2018; Costas-Charvarti et al., 2019). The overall survival rates vary significantly in stage II colon cancer patients, from 66.7% in those patients with stage IIA to 45.7% in those with stage IIC (Gunderson et al., 2010).

In stage II patients who relapse after curative surgery the development of metastasis is the result of occult tumour dissemination prior to treatment. These disseminated tumour cells that remain after curative surgery is termed minimal residual disease (MRD). The presence of MRD depends on two principal factors; firstly the biological characteristics of differing tumour cells within the primary cancer, that is their ability to disseminate, survive in the circulation, implant in distant tissues and survive in their new environment. Secondly the ability of the host immune response to eliminate these disseminated tumour cells. Three groups of MRD can be identified, those patients positive for bone marrow micro-metastasis have an increased risk for delayed relapse, those with circulating tumour cells (CTCs) detected in blood are at high risk of early treatment failure and finally those patients negative for both have a very high 5-year disease free survival (Murray et al., 2020).

The systemic immune-inflammation index (SII) has been proposed to reflect the balance between pro-tumour inflammation and anti-tumour immune function such as T-cytotoxic lymphocytes and thus serve as an index of host immune status (Chen et al., 2017). It is based on the absolute peripheral neutrophil, lymphocyte and platelet counts and calculated using the formula SII=(N x P/L) where N= absolute neutrophil count, P= absolute platelet count and L=absolute lymphocyte count. An elevated SII is the result of either an increased neutrophil and /or platelet count or a decrease in the lymphocyte count. In the published reports a high serum SII was associated with an unfavourable prognosis, a decreased to time to relapse and decreased progression free (PFS) and overall (OS) survival (Chen et al., 2017; Yatabe et al., 2020). 

We present the hypothesis that immune dysfunction as determined by an altered SII is associated with a more aggressive sub-type of MRD and thus is one reason for a worse prognosis. The study was designed to determine if the SII pre and post-surgery was associated with the sub-type of MRD and patient outcome.

## Materials and Methods

A prospective observational single centre study of consecutive patients referred for evaluation of minimal residual disease between January 2007 and December 2014 and who complied with the following inclusion criteria: Pathological Stage II colon cancer, negative surgical margins, negative CT scan of thorax, abdomen and pelvis for metastasis. The study complied with the STROBE guidelines on cohort studies

For each patient, after giving written informed consent, the following were recorded; age, sex, date of surgical treatment, depth of primary tumour invasion (T), and nodal infiltration (N) of the TNM classification (Gunderson et al., 2010). Lympho-vascular and peri-neural infiltration in the primary tumour was recorded as present or absent and the grade of differentiation recorded as well, moderate or poor.

*CTC detection*

Blood samples were collected one month after curative surgery; the first 5ml was discarded to prevent possible contamination by epithelial cells and the second 8ml was collected into tubes containing EDTA (Becton- Vacutainer^®^, USA). The samples were transported at room temperature and processed within 24 hours.

Mononuclear cells were obtained using differential gel centrifugation with Histopaque 1,077^®^ (Sigma-Aldrich, USA) according to the manufacturer’s instructions and re-suspended in 100μl of autologous plasma. 25μl aliquots of cell suspension were used to prepare 4 slides (sialinized, DAKO, USA), air dried for 24 hours and finally fixed using a solution of 70% ethanol, 5% formaldehyde and 25% phosphate buffered saline (PBS) pH 7.4 (DAKO, USA) for five minutes and washed twice with PBS.

The slides were processed within one hour of fixation and incubated with monoclonal anti-CEA clone 11-7 (DAKO, USA) for one hour at room temperature. CTCs were identified using an alkaline phosphatase-anti-alkaline phosphatase based system (LSAB2, DAKO, USA) with neofuschin as the chromogen. Positive samples underwent a second process using anti-CD45 clone 2B11 + PD7/26 (DAKO, USA); incubated for one hour at room temperature and identified using a peroxidase based system (LSAB2, DAKO, USA) with DAB (3,3´diaminobenzadine tetrachloride) as the chromogen.

**Figure 1:**
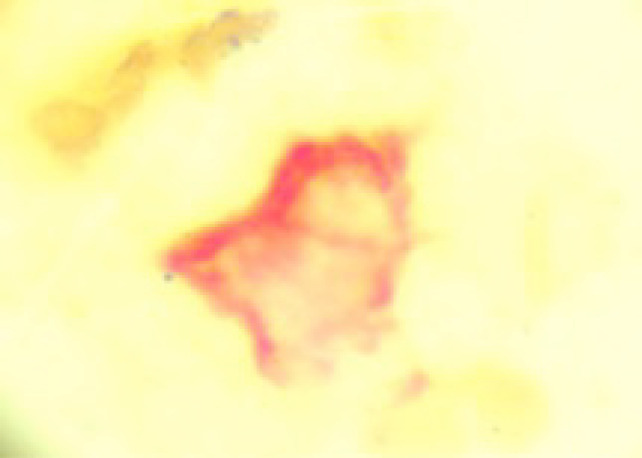
Two CTCs Staining Positive for CEA (red) and Negative for Membrane CD45

**Figure 2:**
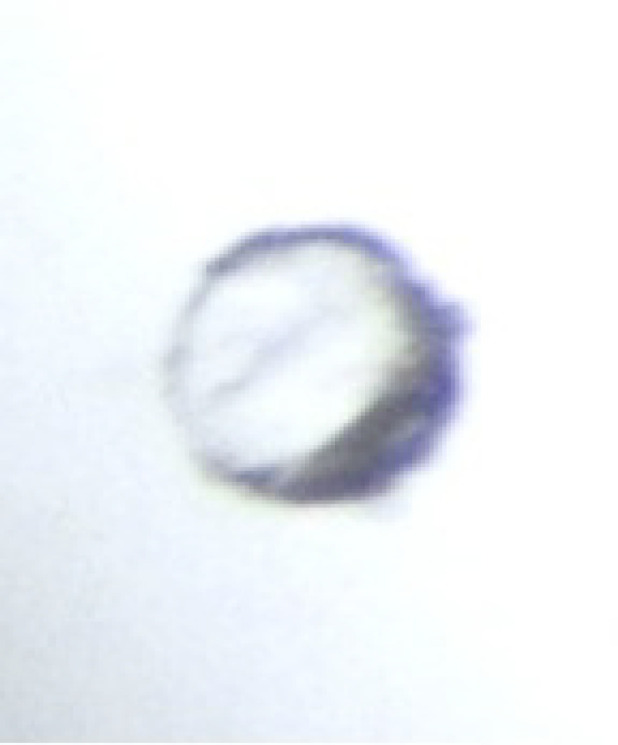
Leukocyte Negative for CEA and Positive for Membrane CD45 (Brown)

A CTC was defined according to the morphological criteria of ISHAGE (Borgen et al., 1999), as a nucleated cell expressing CEA but not CD45. A positive test was defined as the detection of at least one cell/8ml venous blood (Figures 1 and 2).

*Bone marrow micro-metastasis detection*

**Figure 3:**
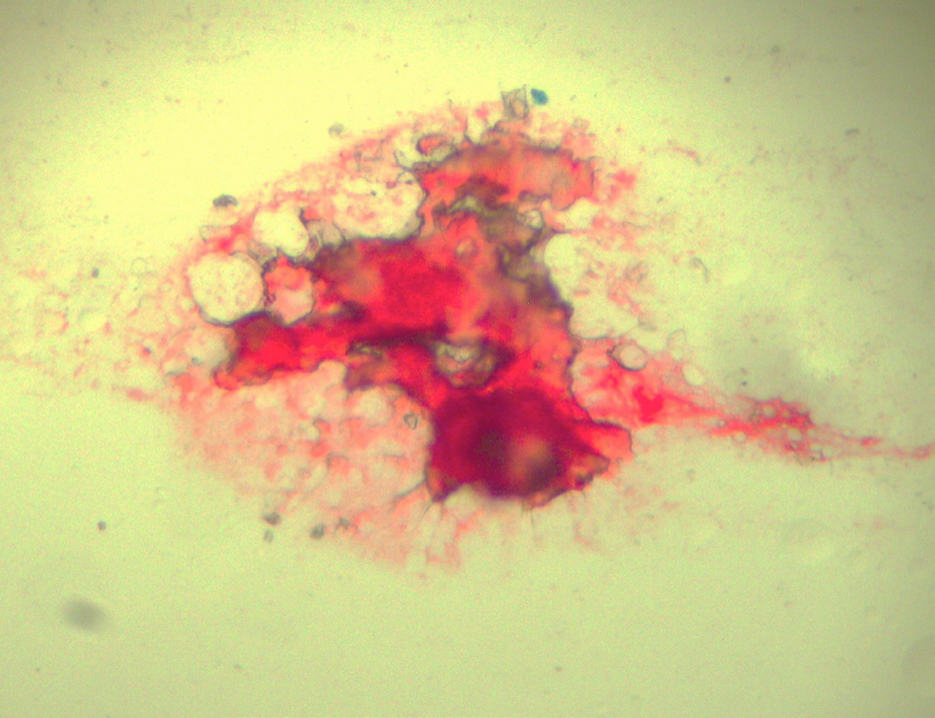
Bone Marrow Micro-Metastasis Staining Positive for CEA (red) and Negative for Membrane CD45

**Figure 4:**
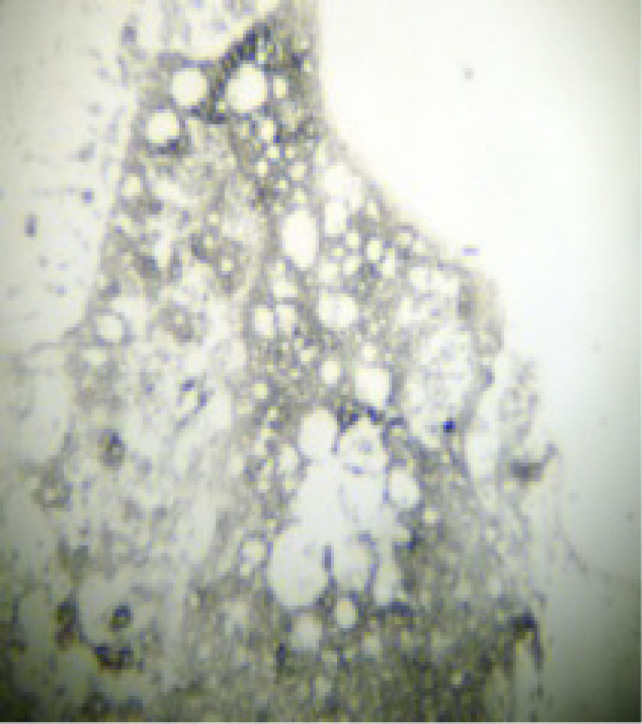
Bone Marrow Staining negative for CEA

Using sedation with intravenous midazolam and anaesthesia local with 2% lidocaine a bone marrow biopsy was taken from the posterior superior iliac crest one month after surgery and the sample used to prepare four ”touch preps” using sialinized slides (DAKO, USA). All four slides were processed as described for CTCs, a micro-metastasis was defined as cells staining positive for CEA and negative for CD45 (Figures 3 and 4).

The patients were divided into three MRD sub-groups according to the presence or absence of CTCs and bone marrow micro-metastasis. Group I negative for both CTCs and micro-metastasis patients; Group II CTC negative, micro-metastasis positive; Group III CTC positive with or without bone marrow micro-metastasis detected.

*Systemic Inflammatory Index*

A full blood count was taken at the same time as the same for CTC analysis and the absolute neutrophil, platelet and lymphocyte counts were determined (Siemens autoanalyzer) and the SII calculated as the absolute neutrophil count x absolute platelet count/absolute lymphocyte count. A cut off value of >700 and >900, based on previously reported studies, was used to analyse the differing parameters (Dong et al,. 2020).

*Follow-up*

Patients were followed up three monthly for the first 2 years, then six monthly until five years. Relapse was defined as a new lesion detected on CT scanning of thorax, abdomen or pelvis. The patients were followed up until 5 years or until relapse, the date of the CT scan detecting relapse was used to define the time to treatment failure. Patients were censured at the time of relapse or after 5 years of disease free progression.

Study end point: The primary study end point was the presence of treatment failure defined as a positive imaging study using CT scanning of the thorax, abdomen and pelvis. The secondary end point was the restricted mean time to failure after primary treatment defined as the time from surgery to the first appearance of an image consistent with metastasis on CT scanning.

*Statistical Analysis*

The analysis was performed using the program Stata (Stata/SE 14.0 for Windows, Stata Corp Lp, 20159), describing according to the nature and distribution of the quantitative and ordinate variables with measurements of central tendency (mean and median) and of dispersion using the inter-quartile range (IQR) and standard deviation (SD). The Shapiro-Wilk Test was used to define the null hypothesis with respect to the normal distribution. The nominal dichotomous variables were described as proportions with their respective confidence intervals.

The three MRD prognostic groups were compared for age, sex, primary tumour differentiation, lymphovascular infiltration, peri-neural infiltration and serum CEA pre-surgery. The Kruskal–Wallis test was used to test whether samples originated from the same distribution and the Pearson’s chi-squared test was used to compare frequencies between MRD sub-groups. A p value <0.05 was taken to signify statistical significance and all tests were two tailed. A SII cut-off value of 700 and 900 was used to determine high and low risk patients and compare with the different clinical-pathological and MRD parameters.

A nonparametric survival analysis was performed at three and five years of follow-up to determine the DFS (Kaplan-Meier) and median DFS of the whole cohort and by MRD sub-groups. The Restricted Mean Survival Time (RMST) for treatment failure was determined for five years of follow-up for the whole cohort and by prognostic group. The RMST establishes the expected time to relapse during the 5-year observation period and its value is the area under the Kaplan-Meier nonparametric survival curve (Royston, Parmar., 2013; A´Hern., 2016). 

Ethical Considerations: The study was approved by the local ethics committee and all patients signed written informed consent.

## Results

One hundred and eighty one patients participated in the study between January 2007 and December 2014; the last follow up was completed in October 2018. The minimum and maximum follow up times were 0.6-5.0 years with a median of 4.0 years (IQR 2.7 years). 82 (46%) of the cohort were male and the median age for the study population was 68 years (IQR 16 years). The median SII was 753 (IQR 551-1135) and 667 (IQR 449-996) pre and post surgery respectively with no significant difference between pre and post-surgical values for the cohort as a whole (p=0.54). 

One hunded and five patients (58%) formed Group I, 36 patients (20%) Group II and 40 patients (22%) Group III. Of the study group 53 (29%) patients relapsed. There were no significant differences between the median SII pre or post-surgery when comparing patients who relapsed with those who did not; 870 (IQR 538-1224) versus 753 (565-1029) (p=0.11) pre-surgery and 919 (IQR 530-1090) versus 618 (380-910) (p=0.72) post-surgery respectively.

*Association of SII and MRD (* Table 1 *)*

There were no significant differences in the median SII when comparing pre and post-surgery values for each MRD sub-group. Nor was there a significant difference in the median SII between the different MRD subgroups pre-surgery. However, one month post-surgery there were significant differences in the SII between MRD subgroups.

Patients in Group III had a significantly higher median SII post surgery than either Group I 947 versus 617 (p=0.04) and Group II 947 versus 687 (p=0.03) respectively. There was no significant difference between Group I and Group II (p=0.73). In Group III patients the number of CTCs detected was positively associated with the SII; the sub-group with 1-2 CTCs/blood sample had a significantly lower median SII compared with patients with ≥ 3 CTCs/blood sample 739 (IQR 634-1081) versus 1084 (IQR 947-2032) respectively (p<0.05). 

*Using a SII >700 as a cut-off value (* Table 1 *)*

There was no significant difference in the frequency of patients with a SII >700 comparing pre and post-surgery values for each positive MRD sub-type (Groups II and III). However in patients MRD negative, the frequency of patients with a SII > 700 significantly decreased after curative surgery from 41% to 27% (p=0.04). The frequency of patients in Group III with a SII >700 was significantly higher both pre and post-surgery when compared to Groups I and II; there was no significant difference between Groups I and II.

*Using a SII > 900 as a cut-off value (* Table 1 *)*

There was no significant difference in the frequency of patients with a SII >900 comparing pre and post-surgery values for each positive MRD sub-type (Groups II and III). However in patients MRD negative, the frequency of patients with a SII > 900 significantly decreased after curative surgery from 35% to 21% (p=0.03). The frequency of patients in Group III with a SII >900 was significantly higher both pre and post-surgery when compared to Groups I and II; there was no significant difference between Groups I and II.

*Disease free progression survival according to MRD sub-type*

**Figure 5:**
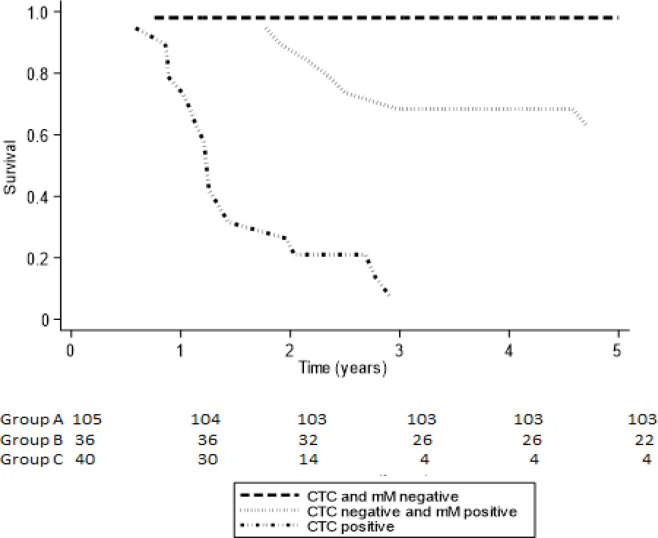
Five Year Progression Free Survival Curves according to Minimal Residual Disease Sub-Type. CTC, circulating tumor cell; mM, micro-metastasis; Observed survival, Kaplan-Meier Survival

Table 2 and Figure 5 show the 5-year observed (Kaplan-Meier) progression free survival (PFS) and the restricted mean progression free survival time at. Patients in Group I had a PFS of 98% with a RMST of 4.9 years to progression; this was superior to patients in Group II who had a PFS of 62.7% and a similar RMST of 4.12 years. Group III patients had a much worse prognosis with a PFS of only 7% and a RMST of 1.7 years. 

**Table 1:** Systemic Inflammatory Index Pre and Post Surgery According to Minimal Residual Disease Subtype

	Group I MRD negative N=105	Group II CTC (-) mM (+) N=36	Group III CTC (+) N=40	p=value
SII Median (IQR)				
Pre-surgery	752 (575-1081)	561 (500-897)	1174 (647-1274)	NS
Post-surgery	617 (380-816)	687 (450-919)	947 (647-12363)	I vs III p=0.04
	p=0.56	p=0.34	p=0.66	II vs III p=0.03
				I vs II p=0.73
Nº CTCs post-surgery				
0			617 (380-816)	0 vs 1-2 p=0.049
1-2			739 (634-1081)	0 vs ≥3 p=0.4
≥ 3			1084 8947-2032)	1-2 vs ≥3 p=0.04
SII >700				
Pre-surgery	43 (41%)	12 (33%)	26 (70%)	I vs II p=0.54ª
				I vs III p=0.003ª
				II vs III p=0.003ª
Post-surgery	28 (27%)	14 (39%)	26 (65%)	I vs II p=0.39ª
	p=0.04ª	p=0.89ª	p=0.81ª	I vs III p=0.01ª
				II vs III p=0.04ª
SII >900				
Pre-surgery	37 (35%	10 (28%)	26 (65%)	I vs II p=0.48ª
				I vs III p=0.003ª
				II vs III p0.003ª
Post-surgery	22 (21%)	12 (33%)	24 (60)	I vs II p=0.20
	p=0.03ª	p=0.79ª	p=0.82ª	I vs III p=0.001ª
				II vs III p=0.03ª

CTC, circulating tumour cell; mM, micro-metastasis; SII, systemic inflammatory index; IQR, inter-quartile range; ª, Pearsons chi squared test; NS, not significant

**Table 2:** Five Tear Observed Progression Free Survival (Kaplan-Meier) and Restricted Mean Survival Time (RMST) According to Minimal Residual Disease Sub-Type

Variable Predictor		% PFS 5 years Kaplan-Meier (95% CI)	Observed RMST 5 years (95% CI)
	I CTC (-) mM (-)	98.1%	4.91 years
	N=105	(87.1-99.7%)	(4.76-5.08)
Prognostic Group			
	II CTC (-) mM (+)	62.7%	4.12 years
	N=36	(37.3-80.2%)	(3.55-4.70)
	III CTC (+)	7.0%	1.71 years
	N=40	(0.5-26.2%)	(1.19-2-23)

%, percentage; CI, confidence interval; CTC, circulating tumour cell; mM, micro-metastasis; RMST is the area under the Kaplan-Meier survival curve determined by the

## Discussion

MRD is the net result of the biological characteristics of the primary tumour cancer cells and the immune response to cancer. The primary tumour not only is the source of disseminated tumour cells but also is responsible for preparing the pre-metastatic niche (Zhao et al., 2018) and modulating the host immune system (Whiteside. 2013). Even in stage IV disease removal of the primary tumour has been reported to increase overall survival and decrease or normalize immune dysfunction (Ahmen et al., 2014; Gulack et al., 2016). In this study of stage II patient’s removal of the primary tumour did not significantly change the SII in the cohort as a whole, nor was there a significant difference in the SII of patients who relapsed compared with those who did not. Pre-surgery there was no significant difference in the SII between MRD sub-groups. This could be explained by the fact that the effect of the primary tumour on the SII was much greater than whatever effect caused by the presence of disseminated tumour cells. 

However, post surgery where the effect of the primary tumour had been eliminated revealed significant differences in the SII between MRD sub-groups. In Group I MRD negative patients the SII significantly decreased following surgical resection, this was not seen in Groups II and III. This suggests that residual micro-metastatic tumour cells are still able to exert an immunosuppressive effect as seen by an unchanged SII. Comparing MRD subtypes with the SII, Group III (CTC positive MRD) patients had a significantly higher SII when compared with MRD negative (Group I) and patients with micro-metastasis positive MRD (Group II). This suggests that the immune dysfunction is significantly worse in CTC positive patients, whereas those with only micro-metastasis had a similar immune function as in MRD negative patients. This was similarly seen in the frequency of patients with a SII higher than 700 and 900. Group III patients had the worse progression free survival and shortest mean time to disease progression. Both a higher SII (Chen et al., 2017; Dong et al,. 2020; Yatabe et al., 2020) and the presence of CTCs ( Rabhari et al., 2010; Tsai et al., 2016) have been reported to be associated with a worse prognosis. An association between immune dysfunction and the number of CTCs has been reported in patients with metastatic breast cancer (Green et al., 2013) but not in patients with colon cancer. The results presented here imply that immune dysfunction and the presence of CTCs is associated, with increasing immune dysfunction being associated with an increased number of CTCs. The resulting immune dysfunction caused the presence of residual tumour cells permitting the escape of CTCs from immune destruction. However, patients with only micro-metastasis had a similar SII as MRD negative patients, this suggests that the biological properties of these tumour cells were unable to produce immune dysfunction as in Group III patients. From the Kaplan-Meier survival curves it can be seen that Group II patients have a similar survival curve as Group I patients for the first two years after surgery. Thus it may not be surprising that one month after surgery the SII values were similar. Although beyond the scope of this study it is possible that with time immune dysfunction increases, as a result of clonal evolution of the tumour cells and thus permits disease progression. Differing from Group I patients, in Group II patients there was no decrease in immune dysfunction as a result of surgical resection, implying that although the micro-metastasis were able to maintain the immune dysfunction it was not sufficient to cause the appearance of CTCs. In breast cancer it has been reported that there is a correlation between the number of disseminated tumour cells detected in bone marrow and T-cell dysfunction, possibly through the production of immunosuppressive cytokines (Campbell et al,. 2005). This immune dysfunction may permit the survival of micro-metastasis without causing disease progression, this is clinically termed dormancy where the patient is asymptomatic and with no evidence of disease but later relapses. In experimental mouse models the ablation of T-lymphocytes accelerates the re-activation and proliferation of disseminated tumour cells and subsequent development of metastasis (Betts et al,. 2012; Eyles et al,. 2010). This may explain why the presence of bone marrow micro-metastasis arte associated with a worse outcome in stage I-III colon cancer (Viehl et al,. 2017).

It is important to acknowledge the limitations of the study; firstly, we used an in house method for the detection of CTCs. The detection of CTCs is method dependent, the frequency of CTCs detected in patients with localized colon cancer using the EpCAM (Epithelial Cell Adhesion Molecule) based CellSearch® system has been reported to be between 5-25% (Tsai et al,. 2012) whereas the size based Metacell® system detected CTCs in 80% of stage II patients (Eliasova et al,. 2017). Comparing flow-cytometry, CellSearch®, quantatitivereal time PCR and cytomorphology, cytomorphology 

showed the least sensitivity and specificity in detecting CTCs, there were no significant differences between the other three methods (Bahnassay et al, 2017). Comparing RT-PCR with CellSearch^®^ CTCs were detected in 75% versus 20% respectively and only 14% using gene mutation analysis (Gervasoni et al,. 2011). The method we used would not detect CEA negative CTCs and bone marrow micro-metastasis but has the advantage of being relatively cheap and although it is 10-100 fold less sensitive than RT-PCR based methods it could be implemented in the routine laboratory of a general hospital. We suggest that the sensitivity may not be a limiting factor, the important question is clinical utility. As yet, independent of the method used to detect tumour cells, there is not an established lower limit of detection with regards to predicting future relapse. Detecting every cancer cell may not be important; patients post allogeneic bone marrow transplantation for leukemia may have very small numbers of leukemic cells detected by RT-PCR in bone marrow samples but remain in remission for many years. Furthermore these leukemia cells may survive for prolonged periods before being eradicated by host defenses (Cross, 1998). As such ultra-sensitive methods to detect tumor cells may over-estimate clinically important minimal residual disease in patients with solid tumours. The method we used was manual and thus inter- and intra-observer variability is higher when compared to automatic or semi-automatic methods. For this reason we used a postive-negative based classification system rather than a cut-off value of x CTCs/sample as used, for example, in the CellSearch^®^ system to limit this potential variability. 

In conclusion, the results of the study suggest that a) the different sub-types of MRD have differing PFS and mean times to treatment failure, corresponding to early and late failure.; b) the severity of immune dysfunction as determined by the SII is associated with differing sub-types of MRD and a worse prognosis; c) increasing immune dysfunction is associated with a more aggressive MRD sub-type, that of CTC positive MRD. Higher numbers of CTCs detected are associated with a more severe immune dysfunction and d) that immune dysfunction has an important role in the type of MRD and prognosis of patients with stage II colon cancer. These results warrant further studies in a larger number of patients.

## Author Contribution Statement

Concept - N.P.M.; Design - N.P.M. RV; Supervision - N.P.M., RV.; Resources - N.P.M.; Materials - N.P.M., RV; Data. Collection and/or Processing – RV, EG, SO,; Analysis and/or Interpretation - N.P.M., RV.,; Literature Search-SO, EG,; Writing Manuscript-NPM, RV,; Critical review-SO, EG,; Final Approval- NPM,; RV,; SO,; EG.

## Data Availability

Due to the Chilean law on patient’s rights the data are not publically available.

## Study Register

The study was not registered in any registering dataset
